# Association of Neighborhood Deprivation With Prostate Cancer and Immune Markers in African American and European American Men

**DOI:** 10.1001/jamanetworkopen.2022.51745

**Published:** 2023-01-20

**Authors:** Margaret S. Pichardo, Tsion Zewdu Minas, Catherine M. Pichardo, Maeve Bailey-Whyte, Wei Tang, Tiffany H. Dorsey, William Wooten, Brid M. Ryan, Christopher A. Loffredo, Stefan Ambs

**Affiliations:** 1 Laboratory of Human Carcinogenesis, National Cancer Institute (NCI), National Institutes of Health (NIH), Bethesda, Maryland; 2 Department of Surgery, Hospital of the University of Pennsylvania, Penn Medicine, Philadelphia; 3 Division of Cancer Control and Population Sciences, NCI, NIH, Rockville, Maryland; 4 School of Medicine, University of Limerick, Limerick, Ireland; 5 Data Science & Artificial Intelligence, R&D, AstraZeneca, Gaithersburg, Maryland; 6 University of Maryland Marlene and Stewart Greenebaum Comprehensive Cancer Center Biostatistics Shared Service, Baltimore; 7 Cancer Prevention and Control Program, Lombardi Comprehensive Cancer Center, Georgetown University Medical Center, Washington, DC

## Abstract

**Importance:**

Neighborhood variables may be factors in the excessive burden of prostate cancer among African American men.

**Objective:**

To examine associations between neighborhood deprivation, circulating immune-oncology markers, and prostate cancer among African American and European American men.

**Design, Setting, and Participants:**

A case-control study was conducted between January 1, 2005, and January 1, 2016. Participants included men with prostate cancer and age- and race-frequency-matched population controls. Participants were recruited at the Baltimore Veterans Affairs Medical Center and University of Maryland Medical Center; controls were obtained through the Maryland Motor Vehicle Administration database. National Death Index follow-up was performed through December 31, 2020, and data analysis was conducted from February 1, 2022, through October 31, 2022.

**Exposures:**

2000 Census-tract Neighborhood Deprivation Index as a standardized score.

**Main Outcomes and Measures:**

Primary outcomes included prostate cancer, all-cause mortality, and disease-specific mortality. Secondary outcomes included the National Comprehensive Cancer Network risk score and serum proteomes for 82 immune-oncology markers with pathway annotation.

**Results:**

Participants included men with prostate cancer (n = 769: 405 African American, 364 European American men) and age- and race-frequency-matched population controls (n = 1023: 479 African American, 544 European American men). The median survival follow-up was 9.70 years (IQR, 5.77 years), with 219 deaths. Among 884 African American men, mean (SD) age at recruitment was 63.8 (7.6) years; mean (SD) age at recruitment among 908 European American men was 66.4 (8.1) years. In the multivariable logistic regression analysis with individual socioeconomic status adjustment, neighborhood deprivation was associated with 55% increased odds of prostate cancer among African American men (odds ratio [OR], 1.55; 95% CI, 1.33-1.81), but was not associated with the disease among European American men. Residing in the most-deprived vs least-deprived neighborhoods corresponded to 88% higher disease odds (OR, 1.88; 95% CI, 1.30-2.75) among all men and an approximate 3-fold increase among African American men (OR, 3.58; 95% CI, 1.72-7.45), but no association was noted among European American men. In Cox proportional hazard regression analyses, socioeconomic status–adjusted neighborhood deprivation was associated with an increased all-cause mortality only among African American men (hazard ratio [HR], 1.28; 95% CI, 1.08-1.53), whereas it was associated with metastatic disease and a 50% increased hazard of a prostate cancer–specific death among all men (HR, 1.50; 95% CI, 1.07-2.09). In analyses restricted to controls, neighborhood deprivation was associated with increased activity scores of serum proteome-defined chemotaxis, inflammation, and tumor immunity suppression.

**Conclusions and Relevance:**

The findings of this study suggest that deprived neighborhood residency may increase the risk of African American men for prostate cancer and a related mortality, potentially through its association with systemic immune function and inflammation.

## Key Points

**Question:**

Is neighborhood deprivation associated with prostate cancer outcomes and circulating immune oncology markers among African American and European American men?

**Findings:**

In this case-control study of 884 African American and 908 European American men in the greater Baltimore area, neighborhood deprivation was associated with increased odds of prostate cancer and a higher risk of all-cause and prostate cancer–specific mortality, and a greater risk for African American men. Neighborhood deprivation was also associated with immune-oncology pathways related to tumor suppression, chemotaxis, and inflammation.

**Meaning:**

The findings of this study suggest that a deprived neighborhood milieu may predispose African American men to lethal prostate cancer, potentially through the systemic immune environment.

## Teaser

This case-control study examines the incidence and mortality of prostate cancer in African American vs European American men based on residence in deprived areas.

## Introduction

Prostate cancer is a leading cause of cancer deaths among African American men.^1^ The built environment and structural racism at the neighborhood level are known determinants of cancer inequities in racially minoritized communities.^2^ Concentrated poverty in African American communities (ie, income- and race-based segregation) is one form of structural racism.^2,3^ It may influence the risk of prostate cancer^4^ and disease prognosis^3^ by direct or indirect effects of social determinants of health, limiting access to medical care,^5,6^ including timely diagnosis, treatment, or by influencing biological processes (ie, increasing allostatic load,^7^ chronic stress signaling,^8,9^ immune function,^10^ and inflammation^11^), or increasing exposure to adverse environments, with reduced opportunities to engage in healthful behaviors.^12,13,14^ Yet, few studies have investigated the association between neighborhood deprivation and prostate cancer^2,4,15,16^ or examined its association with levels of circulating immune-oncology markers.

In this case-control study of African American and European American men, we examined whether neighborhood deprivation is associated with prostate cancer, disease aggressiveness defined by National Comprehensive Cancer Network (NCCN) categorizations, or mortality (all-cause and disease-specific). We also investigated whether neighborhood deprivation is associated with systemic inflammation and immune function.

## Methods

The National Cancer Institute (NCI) Maryland prostate cancer case-control study was conducted among men of self-identified European and African ancestry. Study and eligibility criteria have been previously described.^17,18^ Briefly, between January 1, 2005, and January 1, 2016, the study enrolled a total of 976 men with prostate cancer (489 African American and 487 European American men) who were recruited at the Baltimore Veterans Affairs Medical Center and the University of Maryland Medical Center. Data analysis for this study was conducted from February 1, 2022, through October 31, 2022. Recruitment of men with prostate cancer was by convenience sampling, using the available patient pool, yet with restrictions defined by eligibility. A total of 1034 population controls (486 European American, 548 African American men), frequency matched by age and race, were recruited using the Maryland Department of Motor Vehicle Administration database. At enrollment, trained interviewers administered 2 surveys and collected blood samples. Most of the men with prostate cancer were recruited within 1 year of the disease diagnosis (823 [84%]), with a median of 4.95 months (IQR, 6.74 months) between diagnosis and enrollment for all 976 men. Baseline addresses were geocoded and linked to 2000 census tracts from normalized data derived from the National Neighborhood Change Database produced by Geolytics.^19^ The study was approved by the NCI and University of Maryland Institutional Review Boards. Participants signed an informed consent form and may have received a small compensation not exceeding $50. This study followed the Strengthening the Reporting of Observational Studies in Epidemiology (STROBE) reporting guideline.^20^

### Neighborhood Deprivation

We defined neighborhood deprivation using an approach developed by Messer and colleagues^21^ to operationalize census tract–level socioeconomic deprivation concentration in the 2000 census. However, in deviation from the Neighborhood Deprivation Index (NDI) developed by Messer et al,^21^ which included 20 census variables, we reduced the data to a final set of 6 variables. Based on a study that validated the index in several US states, including Maryland,^22^ a principal components approach was used, where a single factor represented the shared variance from 9 variables representing the socioeconomic status (SES) of study participants. We examined variable loadings and retained those (n = 6) with loading greater than 0.25, without further considering the 95% confidence limit of each loading. Final loadings were examined (eTable 1 in Supplement 1), with the following variables being included in the index: percentage of households in poverty, percentage of female-headed households with dependent children, percentage of households receiving public assistance, percentage of households earning less than $30 000 per year, percentage of males and females unemployed, and percentage of manager occupation. The index was standardized to have a mean (SD) of 0 (1). Lower values indicate lower deprivation, while higher values indicate higher deprivation. The index was operationalized with covariates, as described in the eMethods in Supplement 1, being coded as either continuous score, dichotomized at the median (≤median vs > median), or as quintiles based on its distribution in the population controls.

### Case Definition and Mortality

Incident prostate cancer was defined as being enrolled up to 12 months from diagnosis. Causes of death were identified through the National Death Index database through December 31, 2020. We calculated survival for men with prostate cancer from date of diagnosis to either date of death or censor date of December 31, 2020. Competing events were defined as death from causes other than prostate cancer.

### Statistical Analysis

Initially, we examined the unadjusted association of neighborhood deprivation with either a diagnosis of prostate cancer, using the Mann-Whitney test, or disease risk categorizations, using the Kruskal-Wallis test. We built multivariable logistic regression models and estimated odds ratios (ORs) and 95% CIs for the association between neighborhood deprivation and prostate cancer. Multivariable Cox proportional hazard regression models estimated hazard ratios (HRs) or cause-specific HRs and 95% CI for all-cause and prostate cancer–specific mortality among men with prostate cancer. To evaluate competing risks, we built Fine and Gray models to estimate the subdistribution HR and 95% CI for prostate cancer–specific mortality accounting for competing risks. Multinominal logistic regression models estimated ORs and 95% CIs for the association between neighborhood exposure and NCCN risk scores. We used a stepwise approach to evaluate neighborhood deprivation, beyond individual SES, measured by self-reported educational level and income, on our outcomes of interest, where model 1 adjusted for all covariates minus SES and model 2 adjusted for covariates plus SES. Furthermore, we built separate multivariate analysis of variance (ANOVA) models to simultaneously examine whether neighborhood deprivation estimated the activity scores of 6 biological process/pathways or serum levels of 82 serum proteins that defined these pathways. Exploratory mediation analyses examined the extent to which tumor suppression mediates the association between neighborhood deprivation and prostate cancer mortality. Our analyses were conducted for both the overall study population and separately for African American and European American men. Because of limited observations and events, some analyses for immune-oncology markers and prostate cancer–specific mortality were only conducted with African American and European American men combined. Analyses were conducted in Stata, version 16.1 (StataCorp LLC), and statistical significance was defined as *P* < .05, using unpaired 2-sided testing. Statistical significance in biological processes and pathways was accepted at the Bonferroni-adjusted value of *P* < .05. Robustness was found when significance persisted after adjustments.

Additional information on the census tract analysis and NDI with selection of covariates, mediation analysis, serum protein measurement and pathway annotation, classification of cases using NCCN risk scores, and West African ancestry estimation is available in the eMethods in Supplement 1.

## Results

### Sample Characteristics

Descriptive characteristics of the participants stratified by neighborhood deprivation are reported separately for African American men in eTable 2 and European American men in eTable 3 in Supplement 1. Mean (SD) ages at recruitment were 63.8 (7.6) years in the African American (n = 884) cohort and 66.4 (8.1) years in the European American (n = 908) cohort. The median survival follow-up was 9.70 years (IQR, 5.77 years), with 219 deaths. No significant differences were observed for African American and European American cases and controls by neighborhood deprivation median dichotomization for age at enrollment, prior use of aspirin, a family history of prostate cancer, and a history of type 2 diabetes. In contrast, the NDI was associated with educational level, individual income, body mass index, and smoking status. Among the 2010 men enrolled at baseline, we performed stepwise exclusions for 86 individuals with missing baseline address, 131 nonincident cases (defined as >12 months from diagnosis), and 1 missing body mass index. These exclusions yielded an analytical sample of 1792 men: 769 cases (405 African American, 364 European American) and 1023 controls (479 African American, 544 European American), with 219 all-cause (122 African American, 97 European American) and 59 prostate cancer–specific (36 African American, 23 European American) deaths among cases. Because of missing immune marker data, this secondary analysis was conducted only among 786 control participants. An examination of the clustering pattern by census tract showed that the participants were drawn from across all tracts, limiting any bias.

### Association of NDI With a Prostate Cancer Diagnosis

**Figure 1.**
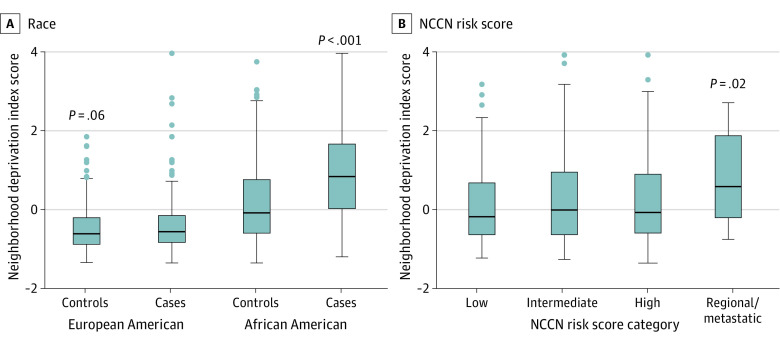
Association of Neighborhood Socioeconomic Deprivation With Prostate Cancer and National Comprehensive Cancer Network (NCCN) Risk Score Categories Neighborhood Deprivation Index scores shown as continuous data for controls and cases (A) and 4 NCCN risk score categories (low to high scores define localized disease) defining the clinical presentation of prostate cancer (B). The error bars represent the 95% CI. Mann-Whitney (A) and Kruskal-Wallis (B) tests were applied for significance testing.

**Figure 2.**
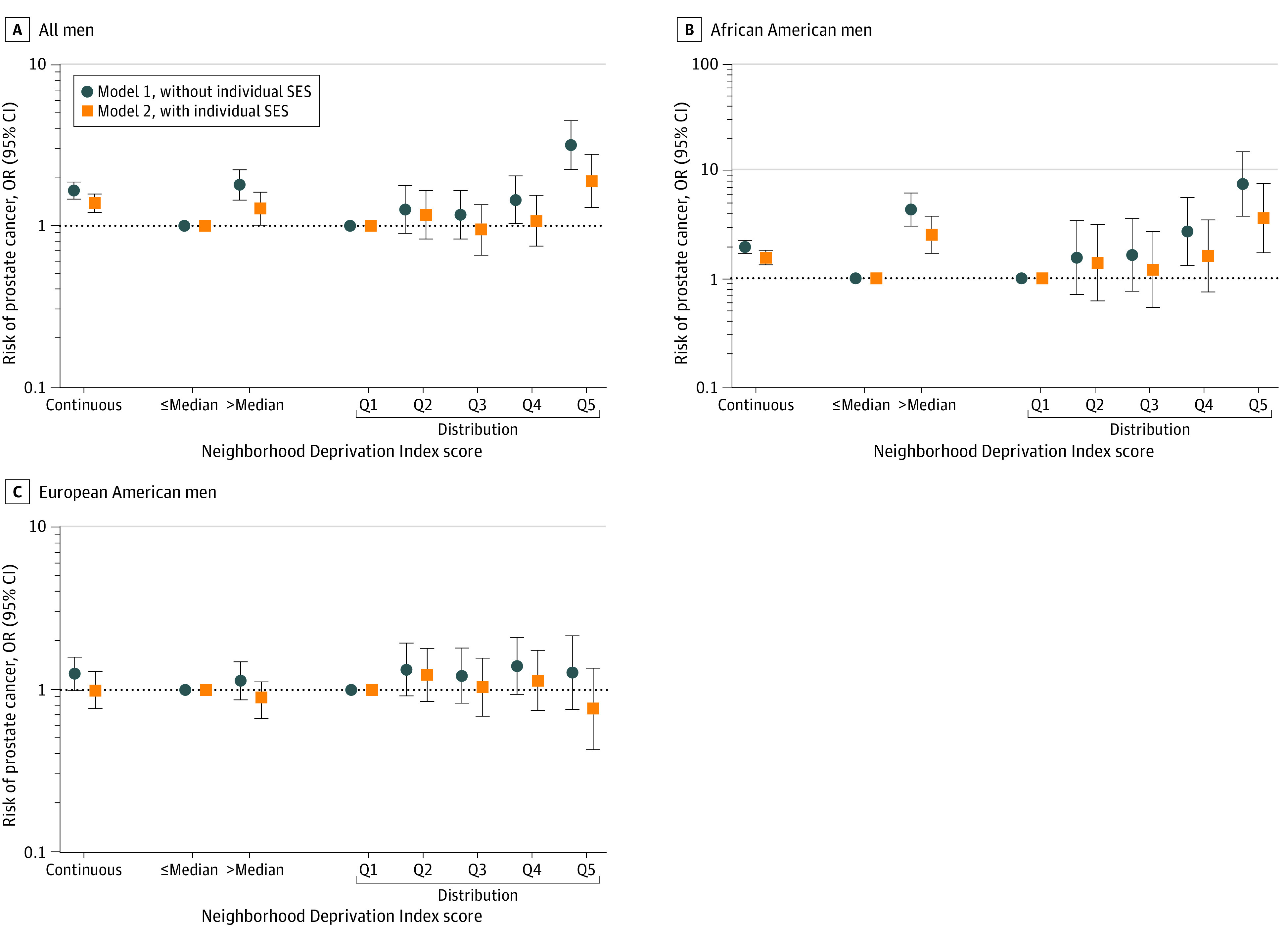
Association of Neighborhood Deprivation With a Diagnosis of Prostate Cancer Odds ratios (ORs) and 95% CIs for the association between Neighborhood Deprivation Index (coded as continuous score, median cutoff, or quintiles [Q]) and a diagnosis of prostate cancer among all men and stratified by self-reported race in all men combined (A), African American men (B), and European American men (C).

Initial analyses of the association between neighborhood deprivation and prostate cancer showed that the deprivation scores were higher for African American than European American men (controls and cases), and highest among African American men with prostate cancer (Figure 1). In multivariable models that combined African American and European American men, neighborhood deprivation was associated with 65% higher odds of prostate cancer (continuous score: OR, 1.65; 95% CI, 1.46-1.86) when not adjusted for SES (model 1 in Figure 2; eTable 4 in Supplement 1). The association was robust and remained significant after adjustment by individual-level SES (model 2 continuous score: OR, 1.38; 95% CI, 1.21-1.57), suggesting that neighborhood factors independent of SES increase prostate cancer risk. Further stratification by race revealed a significant association only for African American men (model 1 continuous score: OR, 1.94; 95% CI, 1.69-2.23; model 2 continuous score: OR, 1.55; 95% CI, 1.33-13 1.81), with evidence of 55% increased odds of prostate cancer among them independent of individual SES, but not for European American men (model 1 continuous score: OR, 1.26; 95% CI, 0.99-1.59; model 2 continuous score: OR, 0.99; 95% CI, 0.77-1.30). When dichotomized at the median, residence in neighborhoods with above-median levels of deprivation was associated with higher odds of a prostate cancer diagnosis for all men and African American men, but not European American men alone, consistent with the findings with the continuous score model (Figure 2, eTable 4 in Supplement 1). When neighborhood deprivation was examined by quintile (Q), only residence in the most-deprived (Q5) vs least-deprived (Q1) areas was associated with 88% increased odds of prostate cancer (model 1: OR, 3.14; 95% CI, 2.22-4.43; model 2: OR, 1.88; 95% CI, 1.30-2.75, for Q5 vs Q1) (Figure 2; eTable 4 in Supplement 1). Stratification by race again revealed an association only among African American men, suggesting an increased cancer risk in the most-deprived areas (model 1: OR, 7.39; 95% CI, 3.73-14.65; model 2: OR, 3.58; 95% CI, 1.72-7.45).

### Association of NDI With NCCN–Defined Risk Groups

Risk scores defined by the NCCN are commonly used in clinical practice for risk assessment of prostate cancer.^23^ We evaluated whether neighborhood deprivation associates with these scores. The data showed that patients with prostate cancer who had developed regional or distant metastatic disease resided in neighborhoods with higher deprivation scores than men with localized disease (Figure 1). In the multivariable analysis, the NDI as a continuous score was consistently associated with elevated odds of regional and distant metastatic disease using model 1 (Table 1), for all men (model 1 continuous score: OR, 1.69; 95% CI, 1.15-2.48), African American men (model 1 continuous score: OR, 1.64; 95% CI, 1.03-2.62), and European American men (model 1 continuous score: OR, 2.06; 95% CI, 1.04-4.06). After additional adjustment by SES (model 2), this association remained at the *P* < .05 significance level among all men (model 2 continuous score: OR, 1.52; 95% CI, 1.01-2.29), but not in the race-stratified analysis (African American men, model 2 continuous score: OR, 1.65; 95% CI, 0.99-2.74; European American men, model 2 continuous score: OR, 1.28; 95% CI, 0.59-2.77). The observations with model 2 indicate that neighborhood deprivation is associated with metastatic disease partially independent of individual-level SES. When we performed the same analysis with dichotomized deprivation scores, residence in neighborhoods with deprivation above the median was associated with metastatic disease among all men. Yet, this association became attenuated when further adjusted by individual-level SES (Table 1). In a follow-up sensitivity analysis that compared the possible association between neighborhood deprivation and localized vs regional and distant metastatic disease, the index was associated with increased odds of metastatic compared with localized disease (reference) among all men (eTable 5 in Supplement 1).

**Table 1.** Association of NDI With NCCN Risk Scores Among African and European American Men With Prostate Cancer^a^

NDI operationalization^b^	OR (95% CI)					
	African American + European American men (n = 769)		African American men only (n = 405)		European American men only (n = 364)	
	Model 1	Model 2	Model 1	Model 2	Model 1	Model 2
Continuous score						
Low	1 [Reference]	1 [Reference]	1 [Reference]	1 [Reference]	1 [Reference]	1 [Reference]
Intermediate	1.07 (0.85-1.35)	1.12 (0.88-1.44)	1.15 (0.88-1.52)	1.24 (0.92-1.68)	0.89 (0.58-1.37)	0.86 (0.54-1.38)
High or very high	1.06 (0.81-1.38)	1.03 (0.77-1.36)	1.19 (0.87-1.62)	1.14 (0.81-1.60)	0.76 (0.44-1.30)	0.72 (0.40-1.29)
Regional/distant metastasis	1.69 (1.15-2.48)	1.52 (1.01-2.29)	1.64 (1.03-2.62)	1.65 (0.99-2.74)	2.06 (1.04-4.06)	1.28 (0.59-2.77)
Above median (referent: below median)						
Low	1 [Reference]	1 [Reference]	1 [Reference]	1 [Reference]	1 [Reference]	1 [Reference]
Intermediate	0.91 (0.58-1.42)	1.04 (0.63-1.72)	1.59 (0.74-3.45)	2.36 (0.99-5.67)	0.69 (0.40-1.18)	0.67 (0.37-1.21)
High or very high	1.07 (0.63-1.82)	1.04 (0.58-1.07)	1.85 (0.73-4.73)	1.97 (0.71-5.47)	0.84 (0.45-1.58)	0.82 (0.41-1.64)
Regional/distant metastasis	4.27 (1.42-12.82)	3.20 (0.97-10.56)	5.52 (0.64-47.51)	5.61 (0.56-56.19)	3.23 (0.92-11.27)	1.86 (0.45-7.66)

Abbreviations: NCCN, National Comprehensive Cancer Network; NDI, national deprivation index; OR, odds ratio.

^a^ Low, intermediate, and high- or very high-risk NCCN categories apply to localized cancers.

^b^ NDI was derived from principal components analysis using 2000 census tract for 4 dimensions of socioeconomic status: educational level, employment, occupation, and poverty, standardized to mean (SD) 0 (1). The index was operationalized as continuous (where higher scores indicate greater deprivation) and dichotomized at the median based on control population cutoffs (≤median vs > median). Model 1 logistic regression analysis adjusted for age at study entry (continuous), aspirin use (yes/no), family history of prostate cancer (first-degree relatives, yes/no), diabetes (yes/no), body mass index at study entry (continuous), self-reported race (not included in stratified analyses, African American, European American), smoking status (current, former, never). Model 2 additionally adjusted for educational level (high school or less, some college, college, professional school, missing), individual income (<$10 000, $10 000-$29 999, $30 000-$59 999, $60 000-$90 000, >$90 000).

### Association Between Neighborhood Deprivation and Serum Proteomics-Defined Biological Pathways

A recent study by several authors of the present study reported that immune-oncology marker profiles differ between men of African and European descent, which was partly explained by ancestry.^24^ To evaluate whether marker profiles are influenced by the neighborhood environment, we examined potential associations with 6 serum proteomics-defined biological pathways based on measurement of these 82 immune-oncology markers. We restricted this examination to the control population to exclude a confounding effect of prostate cancer. Applying multivariate ANOVA models (Table 2), neighborhood deprivation was associated with these pathways, explaining 3.3% to 12.0% of the shared variance in the model (Wilk λ = 0.441; *F* statistic = 10.33; *P* < .001). These findings were robust to inclusion of individual-level SES variables (Wilk λ = 0.395; *F* statistic = 6.74; *P* < .001). Additional univariate ANOVA estimates noted in Table 2 and eFigure 1 in Supplement 1 suggest that chemotaxis (*R*^2^ = 11.96%; *P* = .005), inflammation (*R*^2^ = 5.86%; *P* = .031), and tumor immunity suppression (*R*^2^ = 11.12%; *P* = .018) were the key pathways of this association; however, these observations were sensitive to inclusion of SES variables as only chemotaxis remained associated with the NDI independent of individual-level SES (*R*^2^ = 13.9%; *P* = .008).

**Table 2.** Association Between NDI and Serum Proteome Signatures Defining 6 Biological Processes Among 786 African and European American Population Controls^a^

Characteristic	Model statistics	*R*^2^, %	*F*_1,2_	RMSE	*F* statistic	*P* value	ANOVA β 95% CI)^b^	*P* value
**Model 1 (multivariate ANOVA)**								
Wilk λ	0.441	NA	66.0, 4120.3	NA	10.33	<.001	NA	NA
Pillai trace	0.705	NA	66.0, 4.644.0	NA	9.37	<.001	NA	NA
Lawley-Hotelling trace	0.967	NA	66.0, 4604.0	NA	11.24	<.001	NA	NA
Roy largest root	0.556	NA	11.0, 774.0	NA	39.09	<.001	NA	NA
Pathways								
Autophagy	NA	3.28	NA	0.496	2.388	.007	0.032 (−0.015 to 0.080)	.176
Chemotaxis	NA	11.96	NA	0.351	9.555	<.001	0.048 (0.015 0.081)	.005
Inflammation	NA	5.86	NA	0.353	4.378	<.001	0.037 (0.003 to 0.070)	.031
Promotion	NA	9.90	NA	0.415	7.731	<.001	0.03 (−0.008 to 0.071)	.118
Suppression	NA	11.12	NA	0.333	8.803	<.001	0.038 (0.007 to 0.069)	.018
Vasculature	NA	11.86	NA	0.335	9.465	<.001	0.026 (−0.005 to 0.058)	.104
**Model 2 (multivariate ANOVA)**								
Wilk λ	0.395	NA	114.0, 4388.4	NA	6.74	<.001	NA	NA
Pillai trace	0.805	NA	114.0, 4596.0	NA	6.24	<.001	NA	NA
Lawley-Hotelling trace	1.090	NA	114.0, 4556.0	NA	7.26	<.001	NA	NA
Roy largest root	0.571	NA	766.00	NA	23.01	<.001	NA	NA
Pathways								
Autophagy	NA	4.50	NA	0.496	1.890	.012	0.027 (−0.025 to 0.079)	.306
Chemotaxis	NA	13.9	NA	0.349	6.501	<.001	0.050 (0.013 to 0.086)	.008
Inflammation	NA	7.3	NA	0.352	3.189	<.001	0.031 (−0.006 to 0.067)	.104
Promotion	NA	11.2	NA	0.415	5.067	<.001	0.007 (−0.036 to 0.051)	.737
Suppression	NA	12.4	NA	0.332	5.726	<.001	0.021 (−0.013 to 0.056)	.228
Vasculature	NA	13.0	NA	0.335	6.015	<.001	0.024 (−0.012 to 0.059)	.189

Abbreviations: ANOVA, analysis of variance; NA, not applicable; NDI, national deprivation index; RMSE, root mean square error.

^a^ NDI was derived from principal components analysis using 2000 census tract for 4 dimensions of socioeconomic status: educational level, employment, occupation, and poverty, standardized to mean (SD) 0 (1). The index was operationalized as continuous (where higher scores indicate greater deprivation). Model 1 multiple ANOVA adjusted for age at study entry (continuous), aspirin use (yes/no), family history of prostate cancer (first-degree relatives, yes/no), diabetes (yes/no), body mass index at study entry (continuous), self-reported race (not included in stratified analyses, African American, European American), smoking status (current, former, never), and West African ancestry (continuous). Model 2 additionally adjusted for educational level (high school or less, some college, college, professional school, missing), individual income (<$10 000, $10 000-$29 999, $30 000-$59 999, $60 000-$90 000, >$90 000).

^b^ Represent estimates of each pathway modeled independently.

### Association Between NDI and the Variance of 82 Serum Proteins

To further define the association between neighborhood deprivation and circulating proteins, we assessed the serum levels of the 82 individual immune-oncology markers among population controls. In multivariate ANOVA regression, we found evidence of associations between neighborhood deprivation and the levels of these serum proteins (Wilk λ = 0.0092; *F* statistic = 4.47; *P* < .001). The association remained robust after inclusion of SES factors (Wilk λ = 0.0033; *F* statistic = 2.97; *P* < .001). We examined more comprehensively proteins for which neighborhood explained more than 10% of the variance by applying a series of stepwise linear regressions (eFigure 2 in Supplement 1). In a fully adjusted model that included the genotyped West African ancestry proportion of all participants as a covariate, neighborhood deprivation was associated with 53.0% of the variance for PTN, 52.2% of the variance for CXCL5, and 42.7% of the variance for CXCL1, and between 20.4% and 27.1% of the variance for LAMP3, CD27, DCN, ADGRG1, MMP12, MMP7, and CXCL9 (eTable 6 in Supplement 1).

### Association of NDI With Mortality After a Prostate Cancer Diagnosis

**Figure 3.**
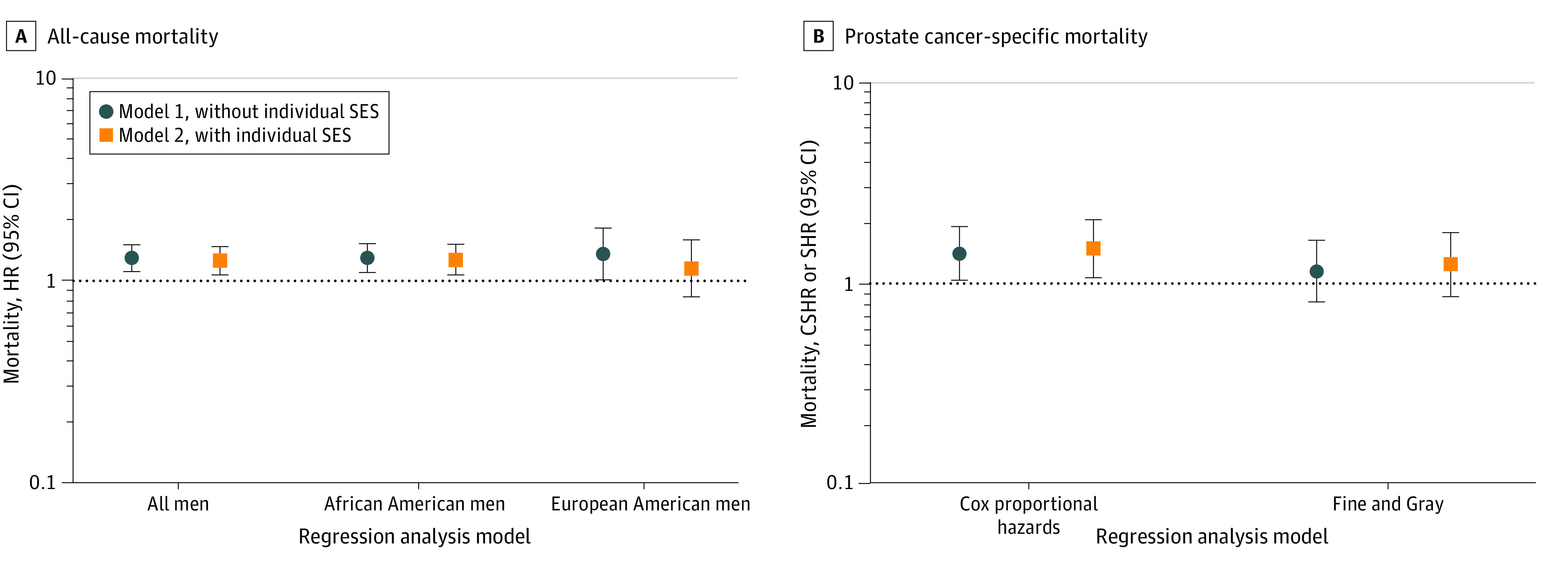
Association of Neighborhood Deprivation With All-Cause and Disease-Specific Mortality Among Men With Prostate Cancer A, All-cause mortality among all men, African American men, and European American men, with hazard ratios (HRs) and 95% CIs. B, Prostate cancer–specific mortality among all men, with cause-specific hazard ratios (CSHRs) using Cox proportional hazards regression or subdistribution hazard ratios (SHRs) and Fine and Gray regression models. Neighborhood Deprivation Index was modeled as a continuous score.

We investigated whether neighborhood deprivation might be a factor in the occurrence of a prostate cancer mortality, using an analysis of all-cause and prostate cancer–specific mortality. Higher deprivation as a continuous score was associated with an SES-adjusted 27% to 28% increased hazard of death from all causes for all men with prostate cancer (model 1: HR, 1.31; 95% CI, 1.12-1.52; model 2: HR, 1.27; 95% CI, 1.08-1.49) and for African American men (model 1: HR, 1.31; 95% CI, 1.11-1.54; model 2: HR, 1.28; 95% CI, 1.08-1.53), but not for European American men (model 1: HR,1.37; 95% CI, 1.02-1.84; model 2: HR, 1.16; 95% CI, 0.84-1.61) (Figure 3; eTable 7 in Supplement 1). Furthermore, NDI was associated with an SES-adjusted 50% increased risk of death from prostate cancer among all men (model 1: cause-specific HR, 1.41; 95% CI, 1.04-1.93; model 2: cause-specific HR, 1.50; 95% CI, 1.07-2.09). Estimates from the secondary analysis using Fine and Gray regressions did not reach statistical significance (Figure 3; eTable 7 in Supplement 1).

Lastly, we performed an exploratory mediation analysis to reveal the extent to which suppression of tumor immunity contributed to the association between neighborhood deprivation and prostate cancer mortality. A previous study linked suppression of tumor immunity to an increased risk of prostate cancer mortality.^24^ In our analysis, we found that suppression of tumor immunity is a candidate mediator of the effect, accounting for 26.2% of the neighborhood deprivation effect on overall survival (*P* = .01) and 18.5% on prostate cancer–specific survival (*P* = .03), with age at diagnosis, body mass index, and race as covariates. With the inclusion of all model 1 covariates, suppression of tumor immunity accounted for 28.3% of the neighborhood deprivation association with overall survival (*P* = .05) and 15.8% on prostate cancer–specific survival (*P* = .27).

## Discussion

In this study, we found that neighborhood socioeconomic deprivation was associated with prostate cancer and related mortalities, but with a more robust association with a prostate cancer diagnosis and all-cause mortality among African American men. Based on our findings, this association might be partly mediated through the suppression of systemic immunity.

Few of the previous studies^25,26^ that investigated neighborhood deprivation and prostate cancer had the diversity of our cohort. Consistent with these and other studies,^15,27^ increased neighborhood deprivation tends to be associated with metastatic and lethal prostate cancer rather than the localized disease. Neighborhood deprivation may exert its detrimental health effects, independent of behavioral risk factors, by increasing systemic inflammation^28,29^ and directly influencing gene expression,^30^ resulting in immune microenvironments that promote tumor development and lethality. One mechanism by which deprivation may exert a biological effect is through increased stress signaling, whereby living in deprived neighborhoods leads to chronic stress and stress-induced proinflammatory signaling and a reduced systemic immune function.^9,10,31^ Stress signaling may contribute to lethal prostate cancer,^8,32^ pointing to the need of further research on how stress and the neighborhood environment may influence oncogenic signaling in one’s body. Furthermore, we found that neighborhood deprivation had the greatest association with a prostate cancer diagnosis and all-cause mortality among African American men. This finding might be explained by prostate cancer–related risk factors, such as chronic stress exposure, that may arise more commonly in neighborhoods with predominantly African American versus European American populations due to discrimination and neighborhood redlining. While preceding studies did not report a differential impact of neighborhood factors comparing African American with European American men, it has been noticed that socioeconomic indicators may show associations with prostate cancer that differ between African American and European American men,^33^ such as neighborhood deprivation and health insurance status.

Previously research^24^ noted that peripheral suppression of tumor immunity and chemotaxis defined by serum proteomics are upregulated in men of African ancestry. Moreover, the suppression of tumor immunity signature was associated with an increased risk of prostate cancer mortality, leading to the hypothesis that environmental and ancestral factors may be associated with increased odds of lethal prostate cancer by enhancing the metastatic process through immune modulation. Therefore, we assessed whether neighborhood deprivation alters the activity of 6 serum proteome-defined pathways and obtained evidence of an association between neighborhood deprivation and the activity scores for peripheral chemotaxis, inflammation, and suppression of tumor immunity. The association with increased chemotaxis was independent of individual-level SES. Notably, chemotaxis is a pathway that has been connected to increased stress-induced metastasis in breast cancer.^34,35^ Our analyses also revealed associations with individual markers, such as CXCL1, CXCL5, and pleiotrophin. The latter has been described as a marker of metastatic prostate cancer.^36^ Similarly, a study using data from the Multi-Ethnic Study of Atherosclerosis^28^ found that higher neighborhood deprivation was associated with higher levels of proinflammatory fibrinogen, interleukin-6, and C-reactive protein, with robust associations for fibrinogen after adjusting for race and SES. Thus, our data are consistent with prior studies. Furthermore, the finding that neighborhood deprivation may enhance peripheral chemotaxis and expression of the metastasis marker pleiotrophin suggests it also may enhance metastasis.

### Strengths and Limitations

As a key strength, the study included approximately equal numbers of African American and European American men as cases and controls, linked to a unique data set including long-term follow-up of all-cause and disease-specific deaths, the measurement of 82 immune-oncology markers with a robust technology, and geocoded baseline data to the census tract level. We used a well-known neighborhood deprivation measure that captures neighborhood SES using 6 census indicators highly relevant for research on structural racism at the neighborhood level.

Limitations of our study include the possibility of a neighborhood selection bias and missing self-reported residential history.^37,38^ Furthermore, we could not examine whether census tract characteristics were associated with individual-level characteristics using our survey. Because we lack participants’ addresses over time, we were unable to examine longitudinal changes, time-varying associations, changes in participant mobility, or the role of deprivation at other known important neighborhood levels (eg, block^26^ or zip code^39^). Furthermore, our study lacks data on prostate cancer screening among participants, which is a factor associated with a prostate cancer diagnosis; therefore, we were not able to adjust for this potential confounder.

## Conclusions

In this case-control study, neighborhood socioeconomic deprivation was associated with a prostate cancer diagnosis and mortality, independent of individual SES. The association between neighborhood deprivation and prostate cancer might be partly mediated through effects that poverty may have on systemic immune-oncology protein expression and activation of signaling pathways related to systemic immune function and inflammation.

## Notes

Supplement 1. **eMethods.** Detailed Methods
eReferences
**eTable 1.** Principal Component Loadings for 2000 Census Tract Neighborhood Deprivation Index Score **eTable 2.** Descriptive Characteristics of African American Men in the NCI-Maryland Case-Control Study, by Neighborhood Deprivation Index Median Dichotomization **eTable 3.** Descriptive Characteristics of European American Men in the NCI-Maryland Case-Control Study, by Neighborhood Deprivation Index Median Dichotomization **eTable 4.** Association of Neighborhood Deprivation Index With a Diagnosis of Prostate Cancer Among African and European American Men **eTable 5.** Association of Neighborhood Deprivation Index With the National Comprehensive Cancer Network Risk Scores Among African and European American Men With Prostate Cancer, Dichotomized as Localized vs Regional/Distant Metastatic Disease **eTable 6.** Proportion of Variance Among Select Immune Oncological Markers Explained by Neighborhood Deprivation Using Independent Stepwise Linear Regression Models, in African American and European American Population Controls **eTable 7.** Association Between Neighborhood Deprivation Index and All-Cause and Disease-Specific Mortality Among African American and European American Men With Prostate Cancer **eFigure 1.** Predictive Margins From Stepwise Regression Models Estimating the Association Between Neighborhood Deprivation Index With (A) Chemotaxis and (B) Inflammation Activity Scores in the Circulation for Population Controls **eFigure 2.** Variance and Residual Plots Showing the Relationship Between Neighborhood Deprivation Index and 82 Immune-Oncology Markers Among the Combined African American and European American Population Controls

Supplement 2.
Data Sharing Statement
