# Supplementary material for: Association of Neighborhood Deprivation With Prostate Cancer and Immune Markers in African American and European American Men
Source: JAMA Netw Open. 2023 Jan 20;6(1):e2251745. doi: 10.1001/jamanetworkopen.2022.51745 (PMC9860532; doi:10.1001/jamanetworkopen.2022.51745)
Supplement: Supplement 1. — eMethods. Detailed Methods eReferences eTable 1. Principal Component Loadings for 2000 Census Tract Neighborhood Deprivation Index Score eTable 2. Descriptive Characteristics of African American Men in the NCI-Maryland Case-Control Study, by Neighborhood Deprivation Index Median Dichotomization eTable 3. Descriptive Characteristics of European American Men in the NCI-Maryland Case-Control Study, by Neighborhood Deprivation Index Median Dichotomization eTable 4. Association of Neighborhood Deprivation Index With a Diagnosis of Prostate Cancer Among African and European American Men eTable 5. Association of Neighborhood Deprivation Index With the National Comprehensive Cancer Network Risk Scores Among African and European American Men With Prostate Cancer, Dichotomized as Localized vs Regional/Distant Metastatic Disease eTable 6. Proportion of Variance Among Select Immune Oncological Markers Explained by Neighborhood Deprivation Using Independent Stepwise Linear Regression Models, in African American and European American Population Controls eTable 7. Association Between Neighborhood Deprivation Index and All-Cause and Disease-Specific Mortality Among African American and European American Men With Prostate Cancer eFigure 1. Predictive Margins From Stepwise Regression Models Estimating the Association Between Neighborhood Deprivation Index With (A) Chemotaxis and (B) Inflammation Activity Scores in the Circulation for Population Controls eFigure 2. Variance and Residual Plots Showing the Relationship Between Neighborhood Deprivation Index and 82 Immune-Oncology Markers Among the Combined African American and European American Population Controls [file jamanetwopen-e2251745-s001.pdf]

Supplementary Online Content

Pichardo MS, Minas TZ, Pichardo CM, et al. Association of neighborhood deprivation with prostate cancer and immune markers in African American and European American men. *JAMA Netw Open*. 2023;6(1):e2251745. doi:10.1001/jamanetworkopen.2022.51745

eMethods. Detailed Methods

eReferences

eTable 1. Principal Component Loadings for 2000 Census Tract Neighborhood Deprivation Index Score

eTable 2. Descriptive Characteristics of African American Men in the NCI-Maryland Case-Control Study, by Neighborhood Deprivation Index Median Dichotomization

eTable 3. Descriptive Characteristics of European American Men in the NCI-Maryland Case-Control Study, by Neighborhood Deprivation Index Median Dichotomization

eTable 4. Association of Neighborhood Deprivation Index With a Diagnosis of Prostate Cancer Among African and European American Men

eTable 5. Association of Neighborhood Deprivation Index With the National Comprehensive Cancer Network Risk Scores Among African and European American Men With Prostate Cancer, Dichotomized as Localized vs Regional/Distant Metastatic Disease

eTable 6. Proportion of Variance Among Select Immune Oncological Markers Explained by Neighborhood Deprivation Using Independent Stepwise Linear Regression Models, in African American and European American Population Controls

eTable 7. Association Between Neighborhood Deprivation Index and All-Cause and Disease-Specific Mortality Among African American and European American Men With Prostate Cancer

eFigure 1. Predictive Margins From Stepwise Regression Models Estimating the Association Between Neighborhood Deprivation Index With (A) Chemotaxis and (B) Inflammation Activity Scores in the Circulation for Population Controls

eFigure 2. Variance and Residual Plots Showing the Relationship Between Neighborhood Deprivation Index and 82 Immune-Oncology Markers Among the Combined African American and European American Population Controls

This supplementary material has been provided by the authors to give readers additional information about their work.

eMethods. Detailed Methods

Census tract analysis. Clustering patterns were examined for each census tract. Over 50% of tracts included one participant, 21% included two participants, 13% included three participants, 6% included four participants, and 5% included five or more participants. Because a low percentage of tracts had 5 or more participants, we did not conduct multilevel modeling to avoid unreliable estimates from increased type 1 error, according to recommendations by Clarke[1].

Neighborhood deprivation index. We applied a principal components approach where a single factor represented the shared variance from nine variables representing socioeconomic standing, including poverty, employment, occupation, and education: percent households in poverty, percent female headed households with dependent children, percent households on public assistance, percent households earning under $30,000/year, percent males and females unemployed, percent manager occupation, percent high school dropout, owner occupied crowded housing and renter occupied crowded housing. We examined variable loadings in the first component and retained those variables loading > 0.25. Loadings were examined and percent households in poverty, percent female headed households with dependent children, percent households on public assistance, percent households earning under $30,000/year, percent males and females unemployed, and percent manager occupation were used in the final calculation to generate the deprivation index (eTable 1). We excluded percent of high school dropout, percent owner occupied crowded housing, and percent renter occupied crowded housing from the final PCA.

Below are variable loadings for each step in the creation of the index:

**Component**	**Eigenvalue**	**Difference**	**Proportion**	**Cumulative**
Component 1	5.01229	3.73424	0.5569	0.5569
**Principal components (eigenvectors)**	**Component 1**			
Percent households in poverty	0.4151			
Percent manager occupation	-0.3358			
percent female headed households with dependent children	0.3875			
Percent households on public assistance	0.3939			
Percent of high school dropout	0.2520			
Percent males and females unemployed	0.3871			
Percent households earning under $30,000/year	0.4051			
Percent Owner occupied crowded housing	0.1085			
Percent Renter occupied crowded housing	0.1430			

© 2023 Pichardo MS et al. *JAMA Network Open.*

Covariates. We adjusted for *a priori* selected variables based on our previous work[2,3] including age at study entry (years), body mass index (BMI, kg/m^2), self-reported race (AA/EA), education (high school or less, some college, college, professional school), income (<$10,000, $10,000-$29,999, $30,000-$59,999, $60,000-$90,000, >$90,000), smoking history (never, former, current), diabetes (yes, no), aspirin use (yes, no), treatment (none, surgery, radiotherapy, hormone, combination), and disease stage defined by the NCCN risk score. Information on these variables was obtained from our survey and medical records. Using a missing indicator, we categorized missing values for education (n = 2), and smoking history (n = 4) and coded as the highest level for each variable.

Multivariate Analysis of Variance (MANOVA). It is the advantage of using a MANOVA that we analyzed multiple dependent variables simultaneously, rather than analyzing multiple dependent variables using multiple ANOVAs, taking into account the inter-correlations between dependent variables[4] and adjusting for multiple comparisons[5]. We used Wilk's lambda (referred to as the U-statistic) to test the impact of neighborhood deprivation. Wilk's lambda can take values ranging between 0 (for large differences in group means) and 1 (no differences in group means). MANOVA models weight coefficients to account for multicollinearity and multiple comparisons. We further examined a select number of the serum proteins where the multivariate variance (R-squared) was >10%, using stepwise linear regressions as follows: Model 1 evaluated association between marker and West African ancestry, model 2 between marker and neighborhood deprivation index, and model 3 included West African ancestry, neighborhood deprivation index and covariates plus SES.

Mediation analysis. The analysis was completed using the med4way command in Stata which is a 4-way decomposition using regression models. This decomposition breaks down the total effect of the exposure on the outcome into components due to mediation alone, to interaction alone, to both mediation and interaction, and to neither mediation nor interaction. Med4way provides standard errors and confidence intervals for the estimated components using the delta method (default). As the exposure variable, the neighborhood deprivation index was dichotomized at the median, to define a referent level for the exposure as required by Stata. As the mediator variable, we coded suppression of tumor immunity as a continuous score variable. We report the combined mediation and interaction effect that suppression of tumor immunity has.

Serum protein measurement. The serum protein measurements were performed as part of a study to evaluate how inflammation may underlie the excessive burden of prostate cancer in men of African Ancestry (DoD award X81XWH1810588). It was the intent of this study to investigate how ancestry and social factors may drive inflammation and influence the immune response, as described[3]. Randomized serum samples from 846 cases and 846 controls were analyzed and assayed using the proprietary multiplex Proximal Extension Assay by Olink Proteomics (Boston), as reported previously[3]. Olink uses a relative quantification unit, Normalized Protein eXpression, which is in a log-2 format (https://www.olink.com). Out of 92 proteins assayed, ten were detected in less than 20% of the samples (IL33, IL35, IL31, Il2, IFN beta, IL13, IL1 alpha, CXCL12, IFN gamma, and TNF), hence the remaining 82 proteins were used for analysis in the present study. Ninety-five percent of the samples passed a stringent quality control (819 cases and 828 controls). The coefficients of variation (CV) among duplicates were <10% for every marker.

Functional annotation and biological process scores. Circulating proteins were evaluated and grouped according to Olink guidelines and summed into pathway/biological process activity scores using mean z-score values derived from their serum levels, as described previously[3]. Z-score values for proteins belonging to the following biological process were calculated for each study participant: suppression of tumor immunity (Th2 response), promotion of tumor immunity (Th1 response),

© 2023 Pichardo MS et al. *JAMA Network Open.*

chemotaxis/trafficking to tumor, autophagy/metabolism, apoptosis/cell killing, vasculature, or inflammation scores. The biological pathways/processes were evaluated as continuous variables.

Classification of cases using National Comprehensive Cancer Network (NCCN) risk scores. Using the 2019 NCCN guidelines for prostate cancer[6], we assigned risk groups to cases based on the patient's TNM stage, Gleason score, Gleason pattern, and PSA level at diagnosis. Risk groups were defined as localized, regional, and metastatic prostate cancer based on clinical parameters at the time of diagnosis. Localized cases were further classified as low, intermediate, high, and very high risk based on the likelihood of their disease to progress to lethal disease according to the NCCN guidelines. Cases with lymph node involvement but no distant metastasis at diagnosis were classified as regional disease while those with distant metastasis at the time of diagnosis were classified as metastatic disease. For the present study, four *a priori* defined categories for risk groups were used: low, intermediate, high/very high, and regional/metastatic[2,3]. Additional sensitivity analyses evaluated disease stage based on dichotomized NCCN risk groups: regional or metastatic vs. localized stage (low, intermediate, high/very high).

West African ancestry estimation for participants in the NCI-Maryland case-control study. As previously described[3], genomic DNA was isolated from buffy coats (DNeasy Blood & Tissue Kit - Qiagen) or mouthwash samples (standard phenol-chloroform technique). Isolated DNA was genotyped for 100 ancestry informative markers using the Sequenom MassARRAY iPLEX platform, as previously described[7]. Single nucleotide polymorphism genotype calls were generated using Sequenom TYPER software. A genotype concordance rate of > 99% was observed for all markers. Admixture estimates for each study participant were calculated using a model-based clustering method as implemented in the program STRUCTURE v2.3. We applied STRUCTURE v2.3 with an admixture model estimating K (number of sub populations) from 2 to 5 with 100 iterations and parental population genotypes from West Africans, Europeans, and Native Americans, yielding three admixture estimations (West African, European, Native American).

© 2023 Pichardo MS et al. *JAMA Network Open.*

eReferences

1. Clarke P. When can group level clustering be ignored? Multilevel models versus single-level models with sparse data. *J Epidemiol Community Health.* 2008;62(8):752-758.
2. Kiely M, Milne GL, Minas TZ, et al. Urinary Thromboxane B2 and Lethal Prostate Cancer in African American Men. *JNCI: Journal of the National Cancer Institute.* 2022;114(1):123-129.
3. Minas TZ, Candia J, Dorsey TH, et al. Serum proteomics links suppression of tumor immunity to ancestry and lethal prostate cancer. *Nature Communications.* 2022;13(1).
4. Huberty CJ, Morris JD. Multivariate analysis versus multiple univariate analyses. *Methodological issues & strategies in clinical research.*1992:351-365.
5. Ramsey PH. Empirical Power of Procedures for Comparing two Groups onp Variables. *Journal of Educational Statistics.* 2016;7(2):139-156.
6. Mohler JL, Antonarakis ES, Armstrong AJ, et al. Prostate Cancer, Version 2.2019, NCCN Clinical Practice Guidelines in Oncology. *J Natl Compr Canc Netw.* 2019;17(5):479-505.
7. Al-Alem U, Rauscher G, Shah E, et al. Association of genetic ancestry with breast cancer in ethnically diverse women from Chicago. *PLoS One.* 2014;9(11):e112916.

© 2023 Pichardo MS et al. *JAMA Network Open.*

eTable 1. Principal Component Loadings for 2000 Census Tract Neighborhood Deprivation Index Score

**Variables**	**Loadings**
Percent of households in poverty	0.435
Percent manager occupation	-0.348
Percent female headed households with dependent children	0.404
Percent households on public assistance	0.417
Percent males and females unemployed	0.410
Percent households earning under $30,000/year	0.428

© 2023 Pichardo MS et al. *JAMA Network Open.*

eTable 2. Descriptive Characteristics of African American Men in the NCI-Maryland Case-Control Study, by Neighborhood Deprivation Index Median Dichotomization

	**African American Cases, n = 405**			**African American controls, n = 479**		
	**Below Median, n = 49**	**Above Median, n = 356**	***P***	**Below Median, n = 178**	**Above Median, n = 301**	***P***
Age, years, mean (SD)	63.6 (7.4)	62.5 (7.3)	0.329	64.8 (7.7)	64.4 (7.9)	0.554
Education, n (%)			0.002			<0.001
High school or less	16 (32.7)	176 (49.4)		39 (21.9)	104 (34.6)	
Some college	16 (32.7)	134 (37.6)		39 (21.9)	103 (34.2)	
College	11 (22.5)	33 (9.3)		49 (27.5)	56 (18.6)	
Professional school	6 (12.2)	12 (3.4)		50 (28.1)	38 (12.6)	
Missing	0 (0.0)	1 (0.28)		1 (0.6)	0 (0.0)	
Income, n (%)			<0.001			<0.001
< $10,000	13 (26.5)	192 (53.9)		8 (4.5)	104 (34.6)	
$10,000-$30,000	6 (12.2)	86 (24.2)		25 (14.0)	80 (26.6)	
$30,000-$60,000	11 (22.5)	29 (8.2)		52 (29.2)	57 (18.9)	
$60,000-$90,000	14 (28.6)	15 (4.2)		74 (41.6)	33 (11.0)	
> $90,000	5 (10.2)	34 (9.6)		19 (10.7)	27 (9.0)	
Aspirin use, n (%)	19 (38.8)	151 (42.4)	0.628	89 (50.0)	158 (52.5)	0.598
Has family history of prostate cancer, n (%)	2 (4.1)	41 (11.5)	0.113	11 (6.2)	20 (6.6)	0.842
Has diabetes history, n (%)	9 (18.4)	110 (30.9)	0.071	51 (28.7)	99 (32.9)	0.334
BMI at enrollment, n (%)	28.8 (4.5)	28.4 (5.3)	0.584	30.0 (5.1)	29.7 (5.7)	0.548
Smoking status, n (%)			0.027			<0.001
Current	18 (36.7)	95 (26.7)		84 (47.2)	98 (32.6)	
Former	23 (46.9)	121 (34.0)		76 (42.7)	120 (39.9)	
Never	8 (16.3)	135 (37.9)		16 (9.0)	82 (27.2)	
Missing	0 (0.0)	5 (1.4)		2 (1.12)	1 (0.33)	
West African ancestry, mean (SD)[1]	0.75 (0.12)	0.76 (0.11)	0.371	0.70 (0.15)	0.73 (0.12)	0.009

© 2023 Pichardo MS et al. *JAMA Network Open.*

Neighborhood deprivation index was derived from principal components analysis using 2000 Census tract for four dimensions of socioeconomic standing: education, employment, occupation, and poverty, standardized to have mean 0 and standard deviation of 1. The index was operationalized as a dichotomous variable at the median based on control population cut offs (≤ median vs. > median). [1]Sample for African American: cases, n = 360, and controls, n = 458.

© 2023 Pichardo MS et al. *JAMA Network Open.*

eTable 3. Descriptive Characteristics of European American Men in the NCI-Maryland Case-Control Study, by Neighborhood Deprivation Index Median Dichotomization

	**European American cases, n = 364**			**European American controls, n = 544**		
	**Below Median, n = 212**	**Above Median, n = 152**	***P***	**Below Median, n = 334**	**Above Median, n = 210**	***P***
Age, years, mean (SD)	66.1 (8.0)	64.8 (7.8)	0.117	66.9 (7.9)	66.8 (8.6)	0.889
Education, n (%)			<0.001			<0.001
High school or less	38 (17.9)	55 (36.2)		40 (12.0)	63 (30.0)	
Some college	43 (20.3)	50 (32.9)		67 (20.1)	56 (26.7)	
College	64 (30.2)	27 (17.8)		101 (30.2)	55 (26.2)	
Professional school	67 (31.6)	20 (13.2)		126 (37.7)	36 (17.1)	
Missing	0 (0.0)	0 (0.0)		0 (0.0)	0 (0.0)	
Income, n (%)			<0.001			<0.001
< $10,000	24 (11.3)	58 (38.2)		17 (5.1)	37 (17.6)	
$10,000-$30,000	35 (16.5)	37 (24.3)		63 (18.9)	64 (30.5)	
$30,000-$60,000	47 (22.2)	24 (15.8)		76 (22.8)	49 (23.3)	
$60,000-$90,000	95 (44.8)	21 (13.8)		159 (47.6)	47 (22.4)	
> $90,000	11 (5.2)	12 (7.9)		19 (5.7)	13 (6.2)	
Aspirin use, n (%)	118 (55.7)	87 (57.2)	0.765	205 (61.4)	131 (62.4)	0.815
Has family history of prostate cancer, n (%)	22 (10.4)	24 (15.8)	0.125	26 (7.8)	14 (6.7)	0.627
Has diabetes history, n (%)	32 (15.1)	27 (17.8)	0.496	53 (15.9)	47 (22.4)	0.056
BMI at enrollment, n (%)	28.0 (4.1)	28.3 (4.6)	0.546	27.7 (4.5)	29.2 (5.7)	0.001
Smoking status, n (%)			0.102			<0.001
Current	90 (42.5)	54 (35.5)		155 (46.4)	68 (32.4)	
Former	95 (44.8)	68 (44.7)		158 (47.3)	104 (49.5)	
Never	25 (11.8)	30 (19.7)		17 (5.1)	36 (17.1)	
Missing	2 (0.9)	0 (0.0)		4 (1.2)	2 (1.0)	
West African ancestry[1]	0.08 (0.09)	0.08 (0.07)	0.809	0.08 (0.09)	0.09 (0.10)	0.325

© 2023 Pichardo MS et al. *JAMA Network Open.*

Neighborhood deprivation index was derived from principal components analysis using 2000 Census tract for four dimensions of socioeconomic standing: education, employment, occupation, and poverty, standardized to have mean 0 and standard deviation of 1. The index was operationalized as a dichotomous variable at the median based on control population cut offs (≤ median vs. > median). [1]Sample for European American: cases, n = 311, and controls, n = 509

© 2023 Pichardo MS et al. *JAMA Network Open.*

eTable 4. Association of Neighborhood Deprivation Index with a Diagnosis of Prostate Cancer among African and European American Men

	AA + EA men, n = 1,792		AA men only, n = 884		EA men only, n = 908	
Neighborhood Deprivation Index Operationalization	Model 1	Model 2	Model 1	Model 2	Model 1	Model 2
Continuous score, OR (95% CI)	1.65 (1.46, 1.86)	1.38 (1.21, 1.57)	1.94 (1.69, 2.23)	1.55 (1.33, 1.81)	1.26 (0.99, 1.59)	0.99 (0.77, 1.30)
Dichotomized, OR (95% CI)						
Below Median	1.00	1.00	1.00	1.00	1.00	1.00
Above Median	1.79 (1.44, 2.21)	1.28 (1.01, 1.61)	4.30 (3.02, 6.11)	2.52 (1.70, 3.73)	1.14 (0.87, 1.49)	0.90 (0.67, 1.21)
Quintiles, OR (95% CI)						
Q1	1.00	1.00	1.00	1.00	1.00	1.00
Q2	1.26 (0.90, 1.77)	1.17 (0.83, 1.65)	1.55 (0.71, 3.39)	1.39 (0.62, 3.14)	1.33 (0.92, 1.94)	1.24 (0.85, 1.80)
Q3	1.17 (0.83, 1.65)	0.95 (0.66, 1.35)	1.64 (0.76, 3.55)	1.20 (0.54, 2.69)	1.22 (0.83, 1.81)	1.04 (0.69, 1.57)
Q4	1.44 (1.03, 2.03)	1.07 (0.75, 1.54)	2.70 (1.31, 5.55)	1.61 (0.75, 3.45)	1.40 (0.94, 2.10)	1.14 (0.75, 1.75)
Q5	3.14 (2.22, 4.43)	1.88 (1.30, 2.75)	7.39 (3.73, 14.65)	3.58 (1.72, 7.45)	1.28 (0.76, 2.15)	0.77 (0.43, 1.36)

Notes. Abbreviations: AA, African American; EA, European American; OR, Odds Ratio; CI, Confidence Interval.
Neighborhood deprivation index was derived from principal components analysis using 2000 Census tract for four dimensions of socioeconomic standing: education, employment, occupation, and poverty, standardized to have mean 0 and standard deviation of 1. The index was operationalized as a continuous variable (where higher scores indicate greater deprivation), dichotomized at the median (≤ median vs. >median) or as quintiles [Q1-Q5 (more deprivation)] based on control population cut offs. **Model 1** logistic regression analysis adjusted for age at study entry (continuous), aspirin use (yes/no), family history of prostate cancer (first-degree relatives, yes/no), diabetes history (yes/no), body mass index at study entry (continuous), self-reported race (not included in stratified analyses, African American, European American), smoking status (current, former, never). **Model 2** additionally adjusted for education (high school or less, some college, college, professional school, missing), individual income (<$10000, $10000-$29999, $30000-$59999, $60000-$90000, >$90000).

© 2023 Pichardo MS et al. *JAMA Network Open.*

eTable 5. Association of Neighborhood Deprivation Index with the National Comprehensive Cancer Network Risk Scores among African and European American Men with Prostate Cancer, Dichotomized as Localized vs. Regional/Distant Metastatic Disease

	AA + EA men, n = 769		AA men only, n = 405		EA men only, n = 364	
Neighborhood Deprivation Index Operationalization	Model 1	Model 2	Model 1	Model 2	Model 1	Model 2
Continuous score, OR (95% CI)						
Localized	1.00	1.00	1.00	1.00	1.00	1.00
Regional/distant metastatic	1.60 (1.14, 2.24)	1.42 (0.99, 2.02)	1.44 (0.96, 2.16)	1.40 (0.91, 2.18)	2.30 (1.24, 4.29)	1.49 (0.74, 3.01)
Above Median (referent: Below Median), OR (95% CI)						
Localized	1.00		1.00	1.00	1.00	1.00
Regional/distant metastatic	4.42 (1.56, 12.57)	3.09 (1.00, 9.58)	3.63 (0.46, 28.75)	2.92 (0.32, 26.28)	4.07 (1.24, 13.39)	2.42 (0.63, 9.33)

Notes. Abbreviations: AA, African American; EA, European American, OR, Odds Ratio; CI, Confidence Interval. Neighborhood deprivation index was derived from principal components analysis using 2000 Census tract for four dimensions of socioeconomic standing: education, employment, occupation, and poverty, standardized to have mean 0 and standard deviation of 1. The index was operationalized as a continuous variable (where higher scores indicate greater deprivation) and dichotomized at the median based on control population cut offs (≤ median vs. >median). **Model 1** logistic regression analysis adjusted for age at study entry (continuous), aspirin use (yes/no), family history of prostate cancer (first-degree relatives, yes/no), diabetes history (yes/no), body mass index at study entry (continuous), self-reported race (not included in stratified analyses, African American, European American), smoking status (current, former, never). **Model 2** additionally adjusted for education (high school or less, some college, college, professional school, missing), individual income (<$10000, $10000-$29999, $30000-$59999, $60000-$90000, >$90000).

© 2023 Pichardo MS et al. *JAMA Network Open.*

eTable 6. Proportion of Variance among Select Immune Oncological Markers Explained by Neighborhood Deprivation using Independent Stepwise Linear Regression Models, in African American and European American Population Controls

	**Model R-squared**		
**Select Immune Oncological Markers**	**Model 1**	**Model 2**	**Model 3**
PTN	10.2%	1.5%	53.0%
CXCL5	49.4%	11.9%	52.2%
CXCL1	40.4%	10.9%	42.7%
CXCL9	4.2%	0.1%	0.271
MMP7	2.6%	1.1%	0.261
MMP12	0.0%	0.7%	0.241
ADGRG1	0.0%	0.1%	0.233
DCN	0.0%	0.2%	0.217
CD27	0.2%	1.7%	0.205
LAMP3	1.2%	0.7%	0.204
PGF	0.9%	0.2%	0.189
TNFRSF12A	0.1%	0.0%	0.183
CCL23	10.8%	1.9%	0.177
MCP2	15.0%	5.0%	0.177
TNFRSF9	2.4%	0.0%	0.175
ANGPT2	0.1%	1.1%	0.170
MCP1	14.3%	2.5%	0.163
NCR1	7.4%	1.3%	0.162
CXCL11	12.7%	3.4%	0.161
TNFRSF4	4.2%	0.5%	0.160
TWEAK	9.1%	2.2%	0.159
CD83	0.6%	0.3%	0.145
TNFRSF21	2.5%	0.5%	0.139
IL12	4.2%	0.8%	0.135
GAL1	0.3%	0.0%	0.133
CX3CL1	0.2%	0.0%	0.114
HGF	1.0%	0.1%	0.113
GAL9	0.0%	0.7%	0.112
CCL20	2.1%	3.4%	0.101

Notes. Immune oncological markers shown were selected from MANOVA models if the proportion of variance explained was greater >10%.
Neighborhood deprivation index was derived from principal components analysis using 2000 Census tract for four dimensions of socioeconomic standing: education, employment, occupation, and poverty, standardized to have mean 0 and standard deviation of 1. The index was operationalized as a continuous variable (where higher scores indicate greater deprivation).
Model 1 examined association between immune oncological marker and West African ancestry (continuous) only.
Model 2 examined association between immune oncological marker and neighborhood deprivation index (continuous).
Model 3 examined fully adjusted association between immune oncological marker, neighborhood deprivation index (continuous), West African ancestry (continuous), and other covariates: age at study entry (continuous), aspirin use (yes/no), family history of prostate cancer (first-degree relatives, yes/no), diabetes history (yes/no), body mass index at study entry (continuous), self-reported race (not included in stratified analyses, African American, European American), smoking status (current, former, never), education (high school or less, some college, college, professional school, missing), individual income (<$10000, $10000-$29999, $30000-$59999, $60000-$90000, >$90000).

© 2023 Pichardo MS et al. *JAMA Network Open.*

eTable 7. Association between Neighborhood Deprivation Index and All-cause and Disease-Specific Mortality among African American and European American Men with Prostate Cancer

	AA + EA men, n = 769		AA men only, n = 405		EA men only, n = 364	
Median survival time, years	5.72		4.86		6.14	
	Model 1	Model 2	Model 1	Model 2	Model 1	Model 2
All-cause mortality, HR (95% CI)	1.31 (1.12, 1.52)	1.27 (1.08, 1.49)	1.31 (1.11, 1.54)	1.28 (1.08, 1.53)	1.37 (1.02, 1.84)	1.16 (0.84, 1.61)
Prostate cancer-specific mortality						
Cox Proportional Hazard Regression, CSHR (95% CI)	1.41 (1.04, 1.93)	1.50 (1.07, 2.09)	--	--	--	--
Fine and Gray Regression, SHR (95% CI)	1.15 (0.81, 1.65)	1.25 (0.86, 1.80)	--	--	--	--

Notes. Abbreviations: AA, African American; EA, European American, HR, Hazard Ratio; SHR, Subdistribution Hazard Ratio, CSHR, Cause-specific Hazard Ratio; CI, Confidence Interval. Neighborhood deprivation index was derived from principal components analysis using 2000 Census tract for four dimensions of socioeconomic standing: education, employment, occupation, and poverty, standardized to have mean 0 and standard deviation of 1. The index was operationalized as a continuous variable (where higher scores indicate greater deprivation). Competing events in Fine and Gray models were defined as other cancers or death. **Model 1** Cox regression analysis adjusted for age at study entry (continuous), aspirin use (yes/no), family history of prostate cancer (first-degree relatives, yes/no), diabetes history (yes/no), body mass index at study entry (continuous), self-reported race (not included in stratified analyses, African American, European American), smoking status (current, former, never), treatment (none, surgery, radiation, hormone, combination, missing), and National Comprehensive Cancer Network risk score categories (low, intermediate, high/very high, regional/metastatic). **Model 2** additionally adjusted for education (high school or less, some college, college, professional school, missing), individual income (<$10000, $10000-$29999, $30000-$59999, $60000-$90000, >$90000).

© 2023 Pichardo MS et al. *JAMA Network Open.*

eFigure 1

A

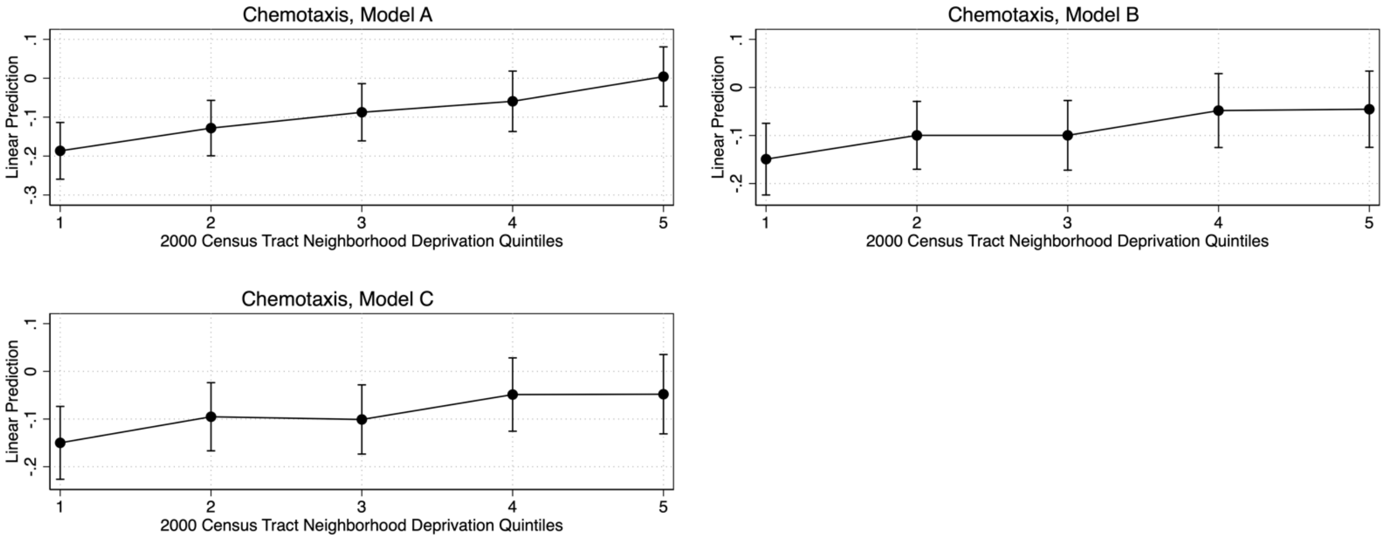

B

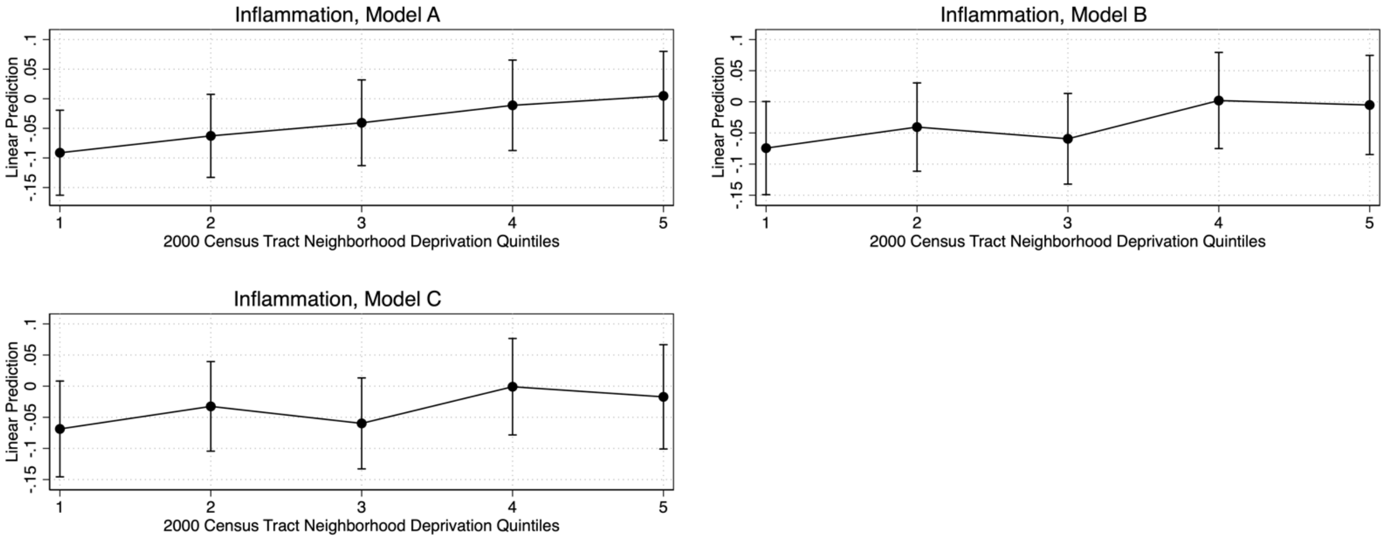

eFigure 1. Predictive margins from stepwise regression models estimating the association between neighborhood deprivation index with (A) chemotaxis and (B) inflammation activity scores in the circulation for population controls. Model A estimates unadjusted associations, model B estimates associations after adjusting for age, aspirin use, family history of prostate cancer, diabetes status, body mass index at study entry, race, smoking, West African ancestry, and model C estimates associations additionally adjusting for socioeconomic status (income and education). The activity scores are derived from serum proteome marker levels as described in methods.

© 2023 Pichardo MS et al. *JAMA Network Open.*

eFigure 2

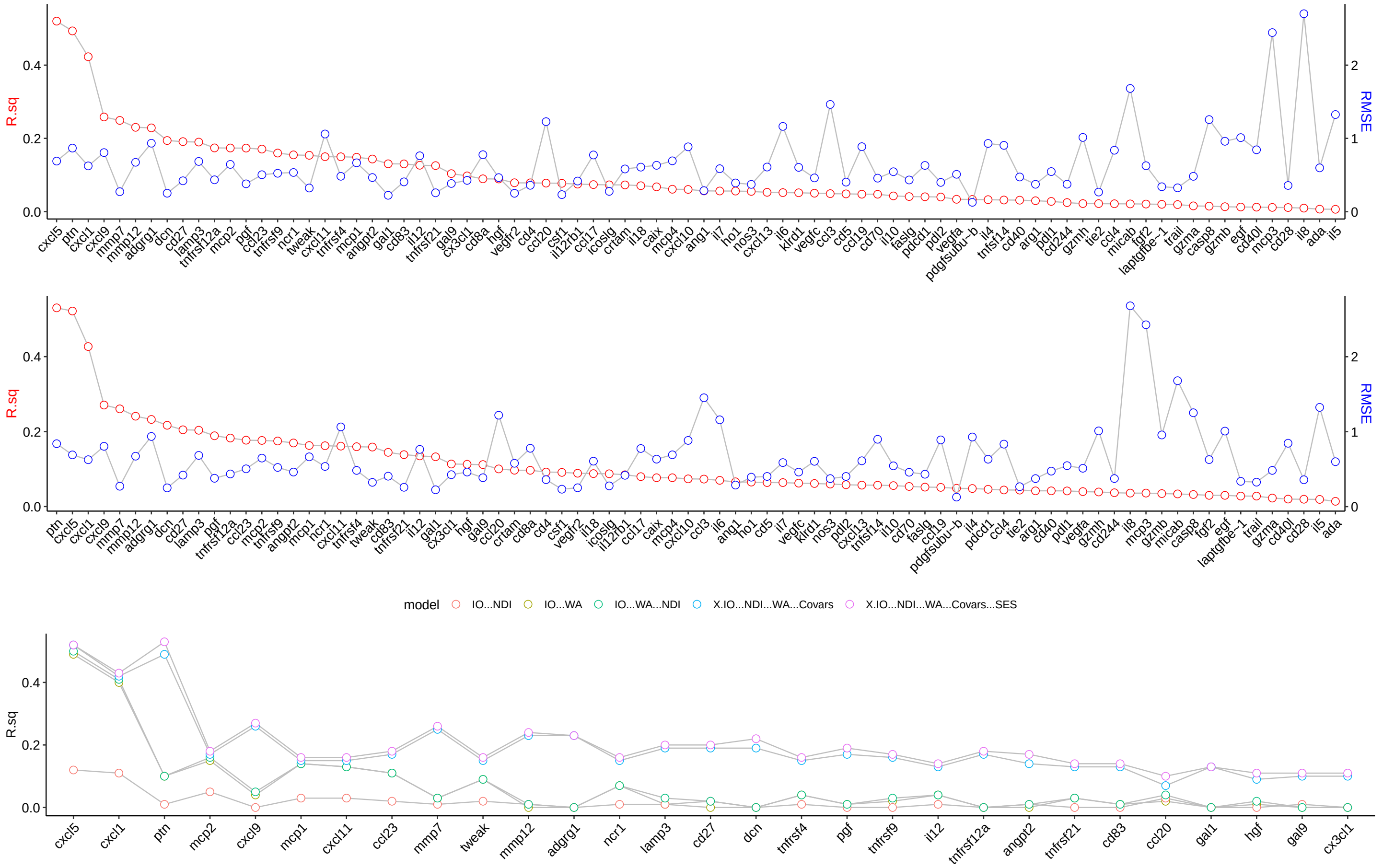

eFigure 2. Variance and residual plots showing the relationship between neighborhood deprivation index and 82 immune-oncology markers among the combined African American and European American population controls. **A)** Graph shows estimates of the deprivation score-explained variance (R-squared) after adjusting for age at study entry (continuous), aspirin use (yes/no), family history of prostate cancer (first-degree relatives, yes/no), diabetes history (yes/no), body mass index at study entry (continuous), self-reported race (not included in stratified analyses, African American, European American), smoking status (current, former, never), treatment (none, surgery, radiation, hormone, combination), and National Comprehensive Cancer Network risk score categories (low, intermediate, high/very high, regional/metastatic). **B)** Graph shows estimates after additionally adjusting for education (high school or less, some college, college, professional school, missing), individual income (<$10,000, $10,000-$29,999, $30,000-$59,999, $60,000-$90,000, >$90,000) as measures of individual SES. **C)** Graph shows variance explained from five independent stepwise linear regression models of select immune oncological markers with: neighborhood socioeconomic deprivation only (model 1), West African ancestry only (model 2), neighborhood socioeconomic deprivation and West African ancestry together (model 3), after adding individual level covariates (model 4), and after additionally adjusting for socioeconomic covariates (model 5).

© 2023 Pichardo MS et al. *JAMA Network Open.*
