# Supplementary material for: Association of Neighborhood Deprivation With Prostate Cancer and Immune Markers in African American and European American Men
Source: JAMA Netw Open. 2023 Jan 20;6(1):e2251745. doi: 10.1001/jamanetworkopen.2022.51745 (PMC9860532; doi:10.1001/jamanetworkopen.2022.51745)
Supplement: Supplement 2. — Data Sharing Statement [file jamanetwopen-e2251745-s002.pdf]

Data Sharing Statement

Pichardo. Association of Neighborhood Deprivation With Prostate Cancer and Immune Markers in African American and European American Men. *JAMA Netw Open*. Published January 20, 2023. doi:10.1001/jamanetworkopen.2022.51745

Data
Data available: No

Additional Information
Explanation for why data not available: We provide the following statement in the manuscript body: Data availability Clinical, demographic, and molecular data (serum proteome data) for the NCI-Maryland prostate cancer study have been deposited at the Open Science Framework at https://doi.org/10.17605/OSF.IO/327HA and as a public GitHub Repository at https://doi.org/10.5281/zenodo.5815262. The remaining data are available within the article, or as supplementary data, or are available from the authors upon request. Personal identifiers such as neighborhood census tract data cannot be shared.
